# Rational Design of Biosafety Level 2-Approved, Multidrug-Resistant Strains of Mycobacterium tuberculosis through Nutrient Auxotrophy

**DOI:** 10.1128/mBio.00938-18

**Published:** 2018-05-29

**Authors:** Catherine Vilchèze, Jacqueline Copeland, Tracy L. Keiser, Torin Weisbrod, Jacqueline Washington, Paras Jain, Adel Malek, Brian Weinrick, William R. Jacobs

**Affiliations:** a Howard Hughes Medical Institute, Department of Microbiology and Immunology, Albert Einstein College of Medicine, Bronx, New York, USA; b Department of Biology and Chemistry, Nyack College, Nyack, New York, USA; Sequella, Inc.

**Keywords:** Mycobacterium tuberculosis, arginine, auxotrophy, methionine, multidrug resistance

## Abstract

Multidrug-resistant (MDR) tuberculosis, defined as tuberculosis resistant to the two first-line drugs isoniazid and rifampin, poses a serious problem for global tuberculosis control strategies. Lack of a safe and convenient model organism hampers progress in combating the spread of MDR strains of Mycobacterium tuberculosis. We reasoned that auxotrophic MDR mutants of *M. tuberculosis* would provide a safe means for studying MDR *M. tuberculosis* without the need for a biosafety level 3 (BSL3) laboratory. Two different sets of triple auxotrophic mutants of *M. tuberculosis* were generated, which were auxotrophic for the nutrients leucine, pantothenate, and arginine or for leucine, pantothenate, and methionine. These triple auxotrophic strains retained their acid-fastness, their ability to generate both a drug persistence phenotype and drug-resistant mutants, and their susceptibility to plaque-forming mycobacterial phages. MDR triple auxotrophic mutants were obtained in a two-step fashion, selecting first for solely isoniazid-resistant or rifampin-resistant mutants. Interestingly, selection for isoniazid-resistant mutants of the methionine auxotroph generated isolates with single point mutations in *katG*, which encodes an isoniazid-activating enzyme, whereas similar selection using the arginine auxotroph yielded isoniazid-resistant mutants with large deletions in the chromosomal region containing *katG*. These *M. tuberculosis* MDR strains were readily sterilized by second-line tuberculosis drugs and failed to kill immunocompromised mice. These strains provide attractive candidates for *M. tuberculosis* biology studies and drug screening outside the BSL3 facility.

## IMPORTANCE

Elimination of Mycobacterium tuberculosis, the bacterium causing tuberculosis, requires enhanced understanding of its biology in order to identify new drugs against drug-susceptible and drug-resistant *M. tuberculosis* as well as uncovering novel pathways that lead to *M. tuberculosis* death. To circumvent the need for a biosafety level 3 (BSL3) laboratory when conducting research on *M. tuberculosis*, we have generated drug-susceptible and drug-resistant triple auxotrophic strains of *M. tuberculosis* suitable for use in a BSL2 laboratory. These strains originate from a double auxotrophic *M. tuberculosis* strain, H37Rv Δ*panCD* Δ*leuCD*, which was reclassified as a BSL2 strain based on its lack of lethality in immunocompromised and immunocompetent mice. A third auxotrophy (methionine or arginine) was introduced via deletion of *metA* or *argB*, respectively, since *M. tuberculosis* Δ*metA* and *M. tuberculosis* Δ*argB* are unable to survive amino acid auxotrophy and infect their host. The resulting triple auxotrophic *M. tuberculosis* strains retained characteristics of *M. tuberculosis* relevant for most types of investigations.

## INTRODUCTION

The World Health Organization (WHO) reported that half a million new tuberculosis (TB) cases in 2017 were multidrug resistant (MDR) (1). Strains of Mycobacterium tuberculosis, the causative agent of TB, are defined as MDR when they are resistant to the two most potent first-line TB drugs, isoniazid (INH) and rifampin (RIF). MDR-TB treatment requires 2 years with second-line TB drugs that have very significant side effects. The TB community is actively pursuing the development of new vaccines and new drugs to reach the WHO goals of eradicating TB by 2050.

Screening for new drugs active against MDR *M. tuberculosis* strains can pose serious issues of worker safety because of the potential aerosolization of these virulent and hard-to-treat bacteria, which require biosafety level 3 (BSL3) containment. These containment requirements limit the number of laboratories that can evaluate new TB drugs or screen existing libraries in a high-throughput manner. Even when BSL3 facilities are available, many institutions do not allow investigators to study MDR *M. tuberculosis* strains for safety reasons. To meet this unmet research need, we have constructed *M. tuberculosis* strains that have been reclassified as BSL2 strains based on attenuation of the virulence achieved by specific deletions of genes involved in *de novo* biosyntheses of various amino acids and vitamins. We and others have demonstrated that *M. tuberculosis* strains that are auxotrophic for amino acids or vitamins failed to grow in mice (2–5). Whereas previous studies showed that the pantothenate auxotroph *M. tuberculosis* H37Rv Δ*panCD* can replicate at low levels in mice (5), the leucine auxotroph *M. tuberculosis* H37Rv Δ*leuCD* does not (2). The double pantothenate-leucine auxotrophic *M. tuberculosis* strain mc^2^6206 (H37Rv Δ*panCD* Δ*leuCD*) was shown to be safer than *Mycobacterium bovis* BCG in immunodeficient mice; mc^2^6206 was therefore approved to be reclassified as a BSL2 strain by the Albert Einstein College of Medicine Institutional Biosafety Committee (6) and many other institutions (personal communications). We reasoned that the addition of two independent, nonreversible auxotrophic mutations would further improve the safety of mc^2^6206. We separately deleted the genes encoding the homoserine *O*-acetyltransferase MetA and the acetylglutamate kinase ArgB in mc^2^6206, leading to methionine and arginine auxotrophs, respectively. Methionine and arginine starvation are bactericidal events in *M. tuberculosis* (7, 8). *M. tuberculosis* H37Rv Δ*metA* and *M. tuberculosis* H37Rv Δ*argB* failed to grow in immunocompetent and immunodeficient mice (7; S. Tiwari, A. V. Tonder, C. Vilchèze, B. Weinrick, V. Mendes, S. E. Thomas, A. Malek, B. Chen, M. Chen, J. Kim, M. Berney, T. L. Blundell, J. Parkhill, and W. R. Jacobs, Jr., submitted for publication). We anticipate that these triple auxotrophic strains could be useful progenitors to construct BSL2-safe, MDR *M. tuberculosis* mutants for safely studying MDR strains and for screening for new TB drugs against MDR *M. tuberculosis*. Interestingly, screening for INH resistance in the arginine triple auxotrophic mutant yielded INH-resistant isolates with large genomic deletions that removed the *katG* gene, involved in INH resistance, and over 44 kb of additional DNA. In this work, we describe the successful construction of BSL2-approved triple auxotrophic antibiotic-sensitive and MDR strains of *M. tuberculosis* and characterize their genetic and phenotypic features *in vitro* and *in vivo*.

## RESULTS

### Construction of BSL2 triple auxotrophic strains.

**FIG 1:**
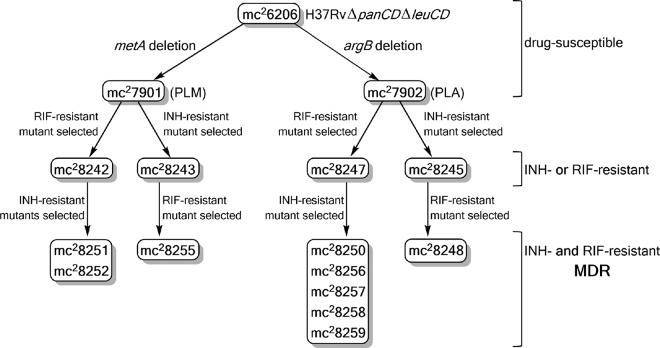
Schematic construction of drug-susceptible and drug-resistant *M. tuberculosis* triple auxotrophs.

The leucine and pantothenate auxotrophic strain mc^2^6206 (Table 1), an *M. tuberculosis* H37Rv strain reclassified as a BSL2 strain (6), was the basis for the construction of triple auxotrophs (Fig. 1). Using a specialized transduction system (6), methionine or arginine auxotrophy was introduced in mc^2^6206 by deleting *metA* (*Rv3341*) or *argB* (*Rv1654*), respectively, The hygromycin cassette selectable marker for the specialized transduction was removed from the knockout strains using γδ resolvase (6), yielding the unmarked pantothenate-leucine-methionine (PLM) auxotroph mc^2^7901 and the unmarked pantothenate-leucine-arginine (PLA) auxotroph mc^2^7902 (Table 1). Whole-genome sequencing of mc^2^7901 and mc^2^7902 confirmed the expected deletions of *metA* and *argB* from mc^2^6206; other mutations were identified by genomic comparison with the laboratory strain *M. tuberculosis* H37Rv. Two mutations were found in both strains: a stop codon to an arginine codon in *Rv0401*, a gene of unknown function, and an L262R mutation in *Rv1272c*, a gene encoding an ABC transporter. These mutations were also present in the parental strain mc^2^6206. Additionally, mc^2^7901 carried a mutation in *Rv2941* (R562G), a gene involved in phthiocerol dimycocerosate biosynthesis, whereas mc^2^7902 had mutations in *Rv2566* (L864P), a gene of unknown function, and the sigma factor A gene *Rv2703* (Q425H).

**TABLE 1:** Bacterial strains

Strain	Genotype	Drug resistance	Reference or source
mc^2^6206	Δ*panCD* Δ*leuCD*		6
mc^2^7271	Δ*panCD* Δ*leuCD* Δ*metA–hyg-sacB*	Hyg^a^	This work
mc^2^7272	Δ*panCD* Δ*leuCD* Δ*argB–hyg-sacB*	Hyg	This work
mc^2^7901	Δ*panCD* Δ*leuCD* Δ*metA*		This work
mc^2^7902	Δ*panCD* Δ*leuCD* Δ*argB*		This work
mc^2^8242	Δ*panCD* Δ*leuCD* Δ*metA rpoB* (H445Y)	RIF	This work
mc^2^8243	Δ*panCD* Δ*leuCD* Δ*metA katG* (W728stop codon)	INH	This work
mc^2^8245	Δ*panCD* Δ*leuCD* Δ*argB* Δ2116169–2162530	INH	This work
mc^2^8247	Δ*panCD* Δ*leuCD* Δ*argB rpoB* (H445Y)	RIF	This work
mc^2^8248	Δ*panCD* Δ*leuCD* Δ*argB* Δ2116169–2162530 *rpoB* (S450L)	INH, RIF	This work
mc^2^8250	Δ*panCD* Δ*leuCD* Δ*argB rpoB* (H445Y) Δ2122397–2170320	INH, RIF	This work
mc^2^8251	Δ*panCD* Δ*leuCD* Δ*metA rpoB* (H445Y) *katG* (S315N)	INH, RIF	This work
mc^2^8252	Δ*panCD* Δ*leuCD* Δ*metA rpoB* (H445Y) *katG* (S315N)	INH, RIF	This work
mc^2^8255	Δ*panCD* Δ*leuCD* Δ*metA katG* (W728stop codon) *rpoB* (S450L)	INH, RIF	This work
mc^2^8256	Δ*panCD* Δ*leuCD* Δ*argB rpoB* (H445Y) *katG* (Δ305–312 bp, frameshift/stop codon)	INH, RIF	This work
mc^2^8257	Δ*panCD* Δ*leuCD* Δ*argB rpoB* (H445Y) *katG* (V1A)	INH, RIF	This work
mc^2^8258	Δ*panCD* Δ*leuCD* Δ*argB rpoB* (H445Y) *katG* (W438R)	INH, RIF	This work
mc^2^8259	Δ*panCD* Δ*leuCD* Δ*argB rpoB* (H445Y) *katG* (W198stop codon)	INH, RIF	This work

a Hyg, hygromycin.

### Characterization of the drug-susceptible, triple auxotrophic strains.

To test the suitability of these triple auxotrophic strains as surrogates for virulent *M. tuberculosis*, *in vitro* growth, acid-fast staining, phage infectibility, and drug susceptibility were assessed. In the experiments described below, a mixture of pantothenate, leucine, arginine, and methionine (PLAM) was added to the growth medium of the triple auxotrophic strains unless otherwise stated.

**FIG 2:**
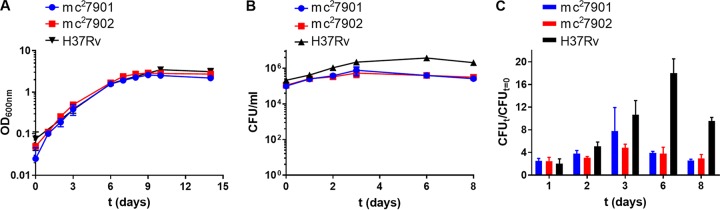
mc^2^7901 and mc^2^7902 grow similarly to virulent *M. tuberculosis in vitro*. (A) Log-phase cultures of mc^2^7901 and mc^2^7902 grown in Middlebrook 7H9-OADC-glycerol-tyloxapol-PLAM were diluted 1/100, and growth was followed by recording optical density at 600 nm (OD_600_) over time. Mean with standard deviation is plotted (*n* = 2). (B) RAW 264.7 macrophages were infected at an MOI of 1 with mc^2^7901, mc^2^7902, or H37Rv. At the indicated time points, macrophages were lysed, and bacterial titers were determined by plating for CFU on Middlebrook 7H10-OADC-glycerol-PLAM plates. PLAM was added to the macrophage growth medium, and the medium was changed at each time point. (C) Growth of mc^2^7901 and mc^2^7902 in RAW 264.7 macrophages relative to the inocula (same experiment as in panel B). Mean with standard deviation is plotted (*n* = 2).

Cultures of mc^2^7901 and mc^2^7902 were first tested in Middlebrook 7H9 medium. mc^2^7901 and mc^2^7902 grew similarly to H37Rv under broth conditions (Fig. 2A). Intracellular growth was evaluated in RAW 264.7 macrophages (Fig. 2B). The triple auxotrophs followed a similar growth pattern as that of H37Rv for the first 2 days of infection. PLA-mc^2^7902 grew more slowly than PLM-mc^2^7901 and H37Rv, but both triple auxotrophs exhibited a 4- to 8-fold increase in CFU (Fig. 2C). Between days 3 and 6 postinfection, the triple auxotrophs stopped growing, and CFU decreased: this phenotype was observed in H37Rv only after day 6 postinfection.

**FIG 3:**
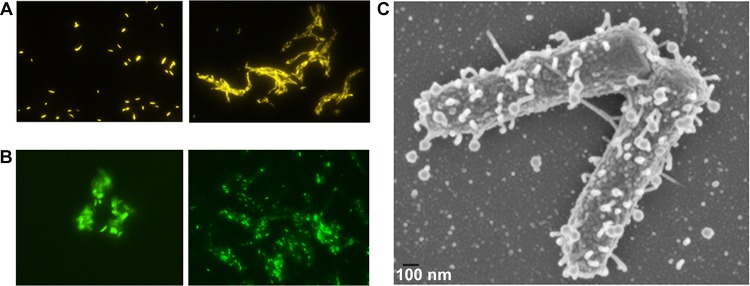
mc^2^7901 and mc^2^7902 retain acid-fastness and susceptibility to phage infection. (A) Acid-fast staining of mc^2^7901 (left) and mc^2^7902 (right) using an auramine kit. Magnification, ×60. (B) Infection of mc^2^7901 (left) and mc^2^7902 (right) cells with phAE912. Magnification, ×60. (C) Scanning electron microscopy of mc^2^7901 (~10^6^ CFU) infected with phAE732 (Φ^2^DRM9, 3 × 10^7^ PFU).

Acid-fast staining has been the basis of clinical diagnosis of TB for over a century. Loss of acid-fastness in *M. tuberculosis* is linked to genetic mutations, alteration in cell wall-associated lipids, lipid accumulation, and persistence (9–11). mc^2^7901 and mc^2^7902 were acid-fast positive (Fig. 3A), indicating that they could be used to study acid-fastness in a BSL2 environment.

Mycobacteriophages (MP) are important tools for mycobacterial genetic studies (12) and TB drug susceptibility testing (13, 14). Through research programs aimed at introducing young students to scientific research (PHIRE and HHMI SEA-PHAGES), thousands of novel mycobacteriophages were discovered using Mycobacterium smegmatis, a nonpathogenic fast-growing mycobacterial species, as the host strain (12). However, many mycobacteriophages that infect M. smegmatis may not infect *M. tuberculosis* (15). The ability of the BSL2-safe *M. tuberculosis* strains mc^2^7901 and mc^2^7902 to serve as mycobacteriophage hosts was therefore examined. The mycobacteriophages phAE912 (16), a DS6A phage restricted to the *M. tuberculosis* complex and expressing mVenus, and Φ^2^DRM9 (17), which infects both M. smegmatis and *M. tuberculosis*, infected mc^2^7901 and mc^2^7902 (Fig. 3B and C). Thus, mc^2^7901 and mc^2^7902 are not restricted for phage infection and phage DNA delivery, demonstrating their suitability as mycobacteriophage hosts and substrates for specialized transduction.

**FIG 4:**
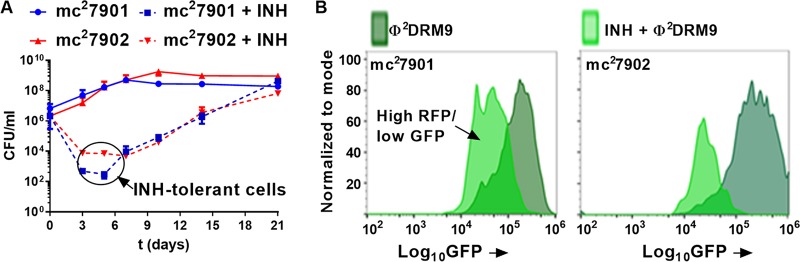
mc^2^7901 and mc^2^7902 generate INH persisters in culture. (A) Log-phase cultures of mc^2^7901 and mc^2^7902 grown in Middlebrook 7H9-OADC-glycerol-tyloxapol-PLAM were diluted 1/100 and treated with INH (1 mg/liter). Samples were taken at the indicated time points, diluted, and plated for CFU. Mean with standard deviation is plotted (*n* = 2). (B) mc^2^7901 and mc^2^7902 cultures treated or not with INH (1 mg/liter) for 2 days were infected with the phage Φ^2^DRM9 and analyzed by flow cytometry. Phage Φ^2^DRM9 contains both the L5 promoter driving GFP (mVenus) expression and the INH persister-specific *dnaK* promoter fused to the red fluorescent protein gene (RFP, tdTomato). The panels show the high-RFP population back-gated for GFP expression, representing the persister population (low GFP/high RFP).

The availability of safe *M. tuberculosis* strains for testing novel therapeutics would circumvent the need for a BSL3 laboratory and allow for high-throughput screening in a BSL2 environment. To test whether triple auxotrophy altered drug susceptibility, the MICs of various first-line and second-line TB drugs were measured (Table 2); mc^2^7901, mc^2^7902, and H37Rv were found equally sensitive to all these drugs. A detailed kinetic analysis of the susceptibility of these triple auxotroph mutants to INH was examined. INH was chosen because it has a very distinctive killing pattern for H37Rv, with rapid death of the bacteria (2- to 3-log decrease in CFU within 4 days). After 4 days of INH treatment, the remaining INH-sensitive bacterial population consists of INH-tolerant cells from which INH-resistant mutants emerge (17). In the presence of INH, mc^2^7901 and mc^2^7902 followed the same death kinetics described above for H37Rv. A rapid decrease in CFU followed by a stabilization of the bacterial population, now consisting of INH-tolerant bacteria, was observed before INH-resistant mutants emerged (Fig. 4A). The presence of an INH-tolerant population was confirmed with the dual green fluorescent protein reporter (GFP)/red fluorescent protein (RFP) reporter phage Φ^2^DRM9 (Fig. 4B) (17). Flow cytometry analysis of INH-treated mc^2^7901 and mc^2^7902 cells infected with Φ^2^DRM9 revealed the presence of a high-RFP/low-GFP population, characteristic of INH persisters (17). Their triple auxotrophy did not impair the ability of the strains to be killed by TB drugs or to produce an INH-tolerant population.

**TABLE 2:** MICs of first-line and second-line TB drugs against the triple auxotrophic strains^a^

Strain	MIC (mg/liter) of TB drug							
	First line		Second line					
	INH	RIF	OF	Km	Moxi	ETH	CFZ	Ami
mc^2^7901	0.06	0.06	0.5	2	0.125	0.625	0.5	0.5
mc^2^8251	1	>4	0.5	4	0.125	0.625	0.5	0.5
mc^2^8255	1	>4	0.5	2	0.125	0.625	0.25	0.5
mc^2^7902	0.06	0.06	0.5	2	0.125	0.625	0.25	0.5
mc^2^8248	>4	>4	0.5	2	0.125	0.625	0.25	0.5
mc^2^8250	>4	>4	0.5	4	0.125	0.625	0.25	0.5
mc^2^8256	>4	>4	0.5	2	0.125	0.625	0.25	0.5
mc^2^8257	>4	>4	0.5	2	0.125	0.625	0.25	0.5
mc^2^8258	>4	>4	0.5	2	0.125	0.625	0.25	0.5
mc^2^8259	>4	>4	0.5	2	0.125	0.625	0.25	0.5

a Abbreviations: Ami, amikacin; CFZ, clofazimine; ETH, ethionamide; INH, isoniazid; Km, kanamycin; Moxi, moxifloxacin; OF, ofloxacin; RIF, rifampin.

Next, we examined whether there is an alteration in the mutation rate in the triple auxotrophs. Following exposure to INH or RIF, mc^2^7901 and mc^2^7902 yielded INH-resistant and RIF-resistant mutants, respectively, at frequencies similar to those found with H37Rv. INH-resistant mutants in mc^2^7901 and mc^2^7902 were isolated at frequencies of 2 × 10^−5^ and 9 × 10^−6^, respectively, compared to 6 × 10^−6^ in H37Rv. RIF-resistant mutants occurred less often than INH-resistant mutants, with frequencies of 4 × 10^−8^, 2 × 10^−7^, and 4 × 10^−8^ for mc^2^7901, mc^2^7902, and H37Rv, respectively. The primary means of resistance to INH and RIF in *M. tuberculosis* are mutations in *katG* (*Rv1908c*), encoding the activator of INH (18), and in *rpoB* (*Rv0667*), encoding an RNA polymerase (19), respectively. Sequence analysis of the *rpoB* and *katG* genes from one drug-resistant isolate from each selection confirmed the presence of an *rpoB* mutation (H445Y) in mc^2^8242 and mc^2^8247, the RIF-resistant mutants of mc^2^7901 and mc^2^7902, and a *katG* mutation in the mc^2^7901-derived, INH-resistant mutant mc^2^8243 (Table 1; Fig. 1). Surprisingly, we could not amplify *katG* from the mc^2^7902-derived, INH-resistant strain mc^2^8245. To characterize mc^2^8245, whole-genome sequencing was performed and revealed a large genomic deletion of 46.3 kbp encompassing *katG* and 50 other genes (Table 3). After the isolation of mc^2^7901 and mc^2^7902 mutants with either INH or RIF monoresistance, we next explored the development of BSL2-safe MDR mutants.

**TABLE 3:** Genes deleted from mc^2^8245 and mc^2^8250

Gene	Coordinates	Cl^a^	Product
*Rv1867*	2115764–2117248	1	Conserved hypothetical protein
*Rv1868*	2117347–2119446	10	Conserved hypothetical protein
*Rv1869c*	2119460–2120695	7	Probable reductase
*Rv1870c*	2120795–2121430	10	Conserved hypothetical protein
*Rv1871c*	2121495–2121884	10	Conserved hypothetical protein
*lldD2*	2121907–2123151	7	Possible l-lactate dehydrogenase (cytochrome)
*Rv1873*	2123174–2123611	10	Conserved hypothetical protein
*Rv1874*	2123684–2124370	10	Hypothetical protein
*Rv1875*	2124381–2124824	10	Conserved hypothetical protein
*bfrA*	2125340–2125819	7	Probable bacterioferritin
*Rv1877*	2125904–2127967	3	Probable conserved integral membrane protein
*glnA3*	2128022–2129374	7	Probable glutamine synthetase
*Rv1879*	2129377–2130513	10	Conserved hypothetical protein
*cyp140*	2130541–2131857	7	Probable cytochrome P450 140
*lppE*	2131907–2132329	3	Possible conserved lipoprotein
*Rv1882c*	2132370–2133203	7	Probable short-chain-type dehydrogenase
*Rv1883c*	2133231–2133692	10	Conserved hypothetical protein
*rpfC*	2133731–2134261	3	Probable resuscitation-promoting factor
*Rv1885c*	2134273–2134872	7	Conserved hypothetical protein
*fbpB*	2134890–2135867	1	Secreted antigen 85-B
*Rv1887*	2136258–2137400	10	Hypothetical protein
*Rv1888c*	2137519–2138079	10	Possible transmembrane protein
*Rv1888A*	2138444–2138617	3	Conserved hypothetical protein
*Rv1889c*	2138661–2139017	10	Conserved hypothetical protein
*Rv1890c*	2139076–2139687	10	Hypothetical protein
*Rv1891*	2139741–2140148	10	Conserved hypothetical protein
*Rv1892*	2140165–2140476	3	Probable membrane proteins
*Rv1893*	2140486–2140704	10	Conserved hypothetical protein
*Rv1894c*	2140739–2141869	10	Conserved hypothetical protein
*Rv1895*	2142521–2143675	7	Possible dehydrogenase
*Rv1896c*	2143535–2144446	10	Conserved hypothetical protein
*Rv1897c*	2144451–2144882	10	Conserved hypothetical protein
*Rv1898*	2144940–2145248	10	Conserved hypothetical protein
*lppD*	2145214–2146245	3	Possible lipoprotein
*lipJ*	2146245–2147633	7	Probable lignin peroxidase
*cinA*	2147662–2148954	0	Probable CinA-like protein
*nanT*	2149006–2150274	3	Probable sialic acid transport membrane proteins
*Rv1903*	2150364–2150768	3	Probable conserved membrane proteins
*Rv1904*	2150954–2151385	10	Conserved hypothetical protein
*aao*	2151433–2152395	7	Probable d-amino acid oxidase
*Rv1906c*	2152425–2152895	10	Conserved hypothetical protein
*Rv1907c*	2153235–2153882	10	Hypothetical protein
*katG*	2153889–2156111	0	Catalase-peroxidase-peroxynitritase T
*furA*	2156149–2156601	9	Ferric uptake regulation protein
*Rv1910c*	2156706–2157299	3	Probable exported protein
*lppC*	2157382–2157987	3	Probable lipoprotein
*fadB5*	2158087–2159091	1	Possible oxidoreductase
*Rv1913*	2159191–2159943	10	Conserved hypothetical protein
*Rv1914c*	2159921–2160328	10	Hypothetical protein
*aceAa*	2160463–2161566	7	Probable isocitrate lyase (first part)
*aceAb*	2161566–2162762	7	Probable isocitrate lyase (second part)
*PPE34*	2162932–2167311	6	PPE family protein
*PPE35*	2167649–2170612	6	PPE family protein

a Cl, classification, based on the TubercuList website (http://genolist.pasteur.fr/TubercuList/), with the following categories: 0, virulence, detoxification, adaptation; 1, lipid metabolism; 3, cell wall and cell processes; 6, PE/PPE; 7, intermediary metabolism and respiration; 9, regulatory proteins; 10, conserved hypothetical proteins.

### Construction of BSL2 triple auxotrophic MDR strains.

The mono-INH- and mono-RIF-resistant mutants mc^2^7901 and mc^2^7902 were used to isolate MDR strains (Fig. 1). INH-resistant mutants were isolated at a frequency of 3 × 10^−7^ from mc^2^8242 (PLM-mc^2^7901 derived, RIF resistant) and 7 × 10^−7^ from mc^2^8247 (PLA-mc^2^7902 derived, RIF resistant), while RIF-resistant mutants were isolated at a frequency of 2 × 10^−8^ from mc^2^8243 (mc^2^7901 derived, INH resistant) and 1 × 10^−8^ from mc^2^8245 (mc^2^7902 derived, INH resistant). The resulting MDR strains were found to be highly resistant to INH and RIF (Table 2).

While *rpoB* and *katG* mutations were identified in most of the MDR mutants (Table 1), selection for INH resistance in mc^2^8247, the RIF-resistant PLA-mc^2^7902 mutant, afforded one MDR mutant, mc^2^8250, from which *katG* could not be amplified by PCR. Whole-genome sequencing of mc^2^8250 identified another large genomic deletion (47.9 kbp) encompassing the *katG* gene (Table 3). The genome of mc^2^8245, the INH-resistant PLA-mc^2^7902 mutant, had a deletion from position 2116169 to 2162530 (Rv1867 to Rv1916), whereas mc^2^8250, the INH- and RIF-resistant PLA-mc^2^7902 mutant, had a deletion from position 2122397 to 2170320 (Rv1872c to Rv1918c). These large deletions contain up to 50 nonessential genes, 37 of them of unknown function (Table 3). In contrast, selecting for INH-resistant mutants in mc^2^8242, the RIF-resistant PLM-mc^2^7901 mutant, led to the isolation of MDR clones having the same mutations in *katG* (S315N) (Table 1).

### Testing of the BSL2 MDR strains *in vitro*.

**FIG 5:**
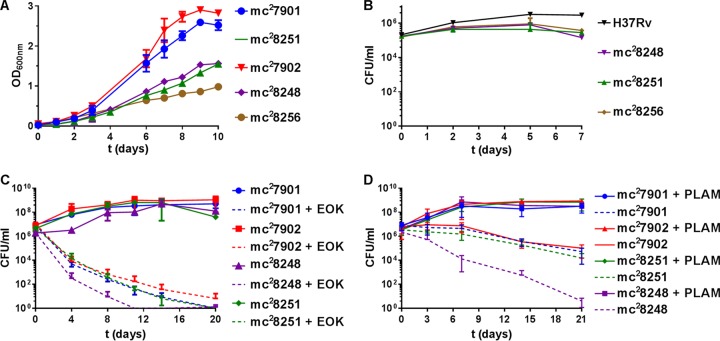
mc^2^7901- and mc^2^7902-derived MDR strains grow slower than parental strains *in vitro* and are killed by second-line TB drugs or nutrient starvation. (A) Log-phase cultures were diluted 1/100, and growth was followed by recording optical density at 600 nm. Mean with standard deviation is plotted (*n* = 2). (B) RAW 264.7 macrophages were infected at an MOI of 1. At the indicated time points, macrophages were lysed to determine bacterial titers. PLAM was added to the macrophage growth medium, and the medium was changed at each time point. (C) PLAM-supplemented log-phase cultures of mc^2^7901, mc^2^7902, mc^2^8248, and mc^2^8251 were treated with EOK (ethionamide [25 mg/liter], ofloxacin [5 mg/liter], kanamycin [20 mg/liter]). (D) Log-phase cultures of mc^2^7901, mc^2^7902, mc^2^8248, and mc^2^8251 were washed five times in PBS and resuspended in Middlebrook 7H9-OADC-glycerol-tyloxapol containing PLAM (dilution factor 1/100) or not. In experiments in panels B, C, and D, the strains were initially grown in Middlebrook 7H9-OADC-glycerol-tyloxapol-PLAM. Samples were taken at the indicated time points, diluted, and plated for CFU on Middlebrook 7H10-OADC-glycerol-PLAM plates. Means with standard deviations are plotted (*n* = 3).

To ensure that these MDR strains could be used to study drug resistance or for drug screening, we examined their *in vitro* growth patterns, drug susceptibility, and safety. In liquid cultures (Fig. 5A) and in macrophages (Fig. 5B), the MDR strains grew slower than their parental strains or virulent *M. tuberculosis* in fully supplemented medium.

Susceptibility testing with second-line TB drugs indicated that the MDR strains were as susceptible as H37Rv to these drugs (Table 2). Next, killing of the MDR strains and that of their parental strains by second-line TB drugs were compared. The triple auxotrophic drug-susceptible parental strains and MDR strains were simultaneously treated with three drugs representing a typical treatment combination for MDR-TB, i.e., one fluoroquinolone (ofloxacin), one injectable drug (kanamycin), and one conventional second-line drug (ethionamide). This combination was efficient in sterilizing the BSL2 drug-susceptible and MDR strains. The MDR PLA-mc^2^8248 strain with a 46-kb genomic deletion was the most susceptible to the treatment, reaching a 6-log decrease in CFU within 10 days (Fig. 5C).

We also examined the susceptibility of these triple auxotrophic strains to nutrient starvation. Starvation of the drug-susceptible triple auxotrophs mc^2^7901 and mc^2^7902 for pantothenate, leucine, arginine, and methionine led to a 2-log reduction in CFU (Fig. 5D). The same result was observed for the PLM-mc^2^7901-derived MDR strain mc^2^8251, but not for the PLA-mc^2^7902-derived mc^2^8248, which had a 6-log reduction in CFU during nutrient starvation (Fig. 5D). These experiments demonstrate that the BSL2 MDR strains lose viability when grown without nutrient supplements or when treated with a second-line TB drug combination.

### Testing the BSL2 MDR strains in mice.

**FIG 6:**
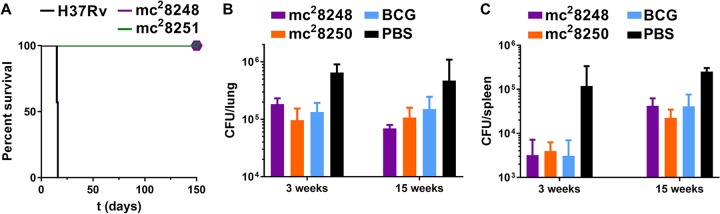
mc^2^7901- and mc^2^7902-derived MDR strains are attenuated in mice and protect against virulent *M. tuberculosis*. (A) Survival of immunocompromised SCID mice (7 mice per group) infected with H37Rv and two MDR strains, mc^2^8248 and mc^2^8251, via tail vein injection at a dose of 5 × 10^5^ CFU. (B and C) Immunocompetent C57BL/6 mice were immunized with mc^2^8248, mc^2^8250, or BCG and challenged with H37Rv (low-dose aerosol infection) 6 weeks later. Mice were euthanized 3 and 15 weeks postchallenge to determine lung (B) and spleen (C) bacterial burden.

Auxotrophic mutants of *M. tuberculosis* have been shown to be avirulent and highly attenuated for growth in immunocompromised SCID mice (20). The safety of these triple auxotrophic MDR strains was assessed in SCID mice. The mice were infected via tail vein injection with a high dose (5 × 10^5^ CFU) of H37Rv, mc^2^8248 (PLA-MDR), and mc^2^8251 (PLM-MDR). Mice infected with H37Rv died by 14 days postinfection. Conversely, mice infected with the MDR strains were still alive 5 months postinfection (Fig. 6A).

Vaccination with single or double auxotrophic *M. tuberculosis* strains offers protection against virulent *M. tuberculosis* challenge as effective as, although not better than, the vaccine strain M. bovis BCG (20). We were curious whether the large deletions (46 to 48 kbp) from the genomes of the MDR strains mc^2^8248 and mc^2^8250 would remove overall immunogenicity and the ability to protect against *M. tuberculosis*. To examine this, immunocompetent (C57BL/6) mice were immunized by subcutaneous injection with 5 × 10^5^ CFU of mc^2^8248, mc^2^8250, or BCG followed by an immunization boost 3 weeks later. Six weeks after the primary immunization, the mice were challenged with a low dose (48 CFU) of virulent H37Rv via the aerosol route. The mice were euthanized at 3 and 15 weeks postchallenge to estimate lung and spleen bacterial burdens. Against H37Rv challenge, mc^2^8248 and mc^2^8250 protected the mice as well as BCG did but not better (Fig. 6B and C). Furthermore, none of the colonies isolated from the lung and spleen homogenates at 3 and 15 weeks postchallenge were auxotrophs for PLA or PLM, suggesting that mc^2^8248 and mc^2^8250 had been cleared by the host.

## DISCUSSION

Studies on the physiology, biochemistry, cell structure, and mycobacteriophage infection of *M. tuberculosis* are hampered by the need for BSL3 containment. Analogous studies on MDR *M. tuberculosis* are even more hazardous due to the difficulty of treating infections with these strains, so we sought to make safe variants of MDR *M. tuberculosis*. The generation of auxotrophic mutants can attenuate virulent isolates of *M. tuberculosis* in defined ways (2–5). The most recognized attenuated mutant of the *M. tuberculosis* complex is BCG, which was first isolated from M. bovis in 1904 and has been used to vaccinate children since 1921 (21). Although BCG can be used in BSL2 laboratories, there are great variations in the many BCG isolates passaged over the years in different laboratories (22–24).

We reasoned that the introduction of a mutation inducing a third auxotrophy in the leucine and pantothenate auxotroph mc^2^6206 would provide an ideal level of safety. First, both the leucine and pantothenate auxotrophies rendered significant attenuation in immunocompromised mice (6), and the deletion of two individual genes from the leucine or pantothenate pathway prevented the reversion or suppression of an auxotrophic phenotype to a prototrophic phenotype. Methionine auxotrophy conferred by *metA* deletion and arginine auxotrophy mediated by *argB* deletion are nonreversible, nonsuppressible, and bactericidal under starvation for their respective auxotrophy, and the strains are fully safe in immunodeficient mice (7; Tiwari et al., submitted). Thus, we reasoned that three independent auxotrophies resulting from well-separated genomic deletions would create an insurmountable barrier to restoration of virulence by reversion, suppression, or complementation with genes obtained from other bacteria. Importantly, the two resulting triple auxotrophic strains mc^2^7901 and mc^2^7902 retained signature *M. tuberculosis* properties such as acid-fast staining, INH tolerance, and susceptibility to diverse mycobacteriophages. Interestingly, both triple auxotrophs can be supplemented for growth in macrophage cultures for two to four generations. These properties could be useful in developing high-throughput screens for defective growth in macrophages as well as in assessing *M. tuberculosis* killing by new drugs.

The generation of MDR versions of mc^2^7901 and mc^2^7902 did not greatly alter *in vitro* growth properties. However, these strains were safe as they failed to kill immunocompromised mice after 5 months. Interestingly, a vaccination experiment showed that they protected as well as BCG against virulent *M. tuberculosis*. Thus, we propose that the INH- and/or RIF-resistant version of these triple auxotrophic strains could be used for diverse physiological studies, including assaying the bactericidal properties of novel TB drugs. The discovery and development of new drugs targeting *M. tuberculosis* and particularly drug-resistant *M. tuberculosis* strains are difficult processes encumbered by BSL3 laboratory requirements. However, our triple auxotrophic MDR strains can be used for large chemical library screenings without the need for BSL3 containment, which will reduce the cost of drug screening and encourage other laboratories to reproduce findings.

Surprisingly, two independent, large (46- to 48-kbp) deletions in the PLA auxotrophs were isolated while selecting for INH-resistant mutants using two different parental strains (mc^2^7902 and mc^2^8245). It is worth noting that these deletions were obtained only in strains carrying the *argB* deletion. The INH-resistant mutants isolated from the PLM auxotroph mc^2^7901 carried point mutations in *katG*. Deletions in *katG* (either partial or total) are found in INH-resistant clinical isolates (25), and large genomic deletions (from 2 to 34 kbp, containing *katG* and/or the *furA* gene) were identified in a study of six INH-resistant clinical isolates from Japanese patients (26). The largest deletion identified in the Japanese study extended from genomic position 2130514 to 2164879, a region that overlaps the two deletions in mc^2^8248 and mc^2^8250. We hypothesize that the *argB* deletion or arginine supplementation might favor the formation of these large deletions. *De novo* arginine biosynthesis involves the acetylglutamate kinase ArgB and several other genes, but no genes of the arginine biosynthetic pathway were part of the 46- to 48-kbp deletions. This selectivity for deletion suggests that INH killing and arginine auxotrophy have some heretofore undiscovered commonality.

Arginine or methionine starvation of *M. tuberculosis* lacking *argB* or *metA*, respectively, is a bactericidal event leading to a 3- to 4-log reduction in CFU within 10 days (7; Tiwari et al., submitted), while starving *M. tuberculosis* Δ*leuCD* or *M. tuberculosis* Δ*panCD* for leucine or pantothenate, respectively, for 10 days resulted in no loss of viability (7). Nutrient starvation of the PLM strain mc^2^7901 and the PLA strain mc^2^7902 was static for the first 10 days before becoming bactericidal, suggesting that the static phenotype of leucine or pantothenate starvation is dominant compared to the bactericidal effect of arginine or methionine starvation. On the other hand, nutrient starvation of mc^2^8248, the PLA-MDR strain with the 46-kbp deletion, resulted in sterilization of the strain with similar killing kinetics as those found with *M. tuberculosis* Δ*metA* and *M. tuberculosis* Δ*argB*. It is possible that among the 50 deleted genes, the majority of unknown function, one or more genes exist that would antagonize the static effect of the leucine and pantothenate starvation. It is unlikely, though, that this sterilization upon nutrient starvation is due to the *katG* or *rpoB* mutation, since this nutrient starvation sterilization was not observed in mc^2^8251, the PLM-MDR strain (Fig. 5D).

In conclusion, these triple auxotrophic strains are not only valuable to research laboratories but could also be important tools for clinical and microbiology laboratories as training tools since they retain most of the properties of the tubercle bacillus except virulence. We anticipate that these strains will reveal new discoveries about the biology of *M. tuberculosis*.

## MATERIALS AND METHODS

### Bacterial strains and reagents.

The *M. tuberculosis* strains mc^2^6206 (H37Rv Δ*panCD* Δ*leuCD*) and H37Rv and the M. bovis BCG Danish strain were obtained from laboratory stocks. The strains were grown in Middlebrook 7H9 (Difco, Sparks, MD) supplemented with 10% (vol/vol) oleic acid-albumin-dextrose-catalase (OADC; Difco), 0.2% (vol/vol) glycerol, and 0.05% (vol/vol) tyloxapol at 37°C with shaking. Middlebrook 7H10 (Difco) supplemented with 10% (vol/vol) OADC and 0.2% (vol/vol) glycerol was used as solid medium. PLAM nutrient supplements were used at the following concentrations: l-pantothenate, 24 mg/liter; l-leucine, 50 mg/liter; l-arginine, 200 mg/liter; and l-methionine, 50 mg/liter. Plates were incubated at 37°C for 4 to 8 weeks. The plasmid pYUB1471 (6), shuttle phasmid phAE159 (27), and phage phAE280 (6) were obtained from laboratory stocks. Hygromycin (Gold Biotechnology, St. Louis, MO) was used at concentrations of 50 mg/liter for mycobacteria and 150 mg/liter for Escherichia coli. Phosphate-buffered saline (PBS) was obtained from Corning Cellgro (Manassas, VA). All other chemicals were obtained from Sigma-Aldrich or Thermo (Fisher) Scientific.

### Construction and unmarking of mc^2^7901 and mc^2^7902.

Deletion of *metA* or *argB* in mc^2^6206 was carried out by specialized transduction (6). The transductants were selected on plates containing hygromycin as the selective marker and either methionine or arginine. The hygromycin cassette was excised from the knockout strains using the phage phAE280 and sucrose selection (6). The deletion and unmarked strains were confirmed by PCR and by whole-genome sequencing performed on a MiSeq instrument (Illumina, San Diego, CA).

### Murine macrophage infection.

RAW 264.7 murine macrophages were obtained from laboratory stocks; subcultured in Dulbecco’s modified Eagle’s medium (DMEM; Invitrogen, Carlsbad, CA) supplemented with 10% fetal bovine serum (FBS; Invitrogen), penicillin (100 units), and streptomycin (100 mg/liter); and grown to confluence. Macrophages were seeded into 24-well tissue culture plates at a concentration of ~5 × 10^5^ cells per well and incubated at 37°C overnight for adherence. The *M. tuberculosis* strains were grown to an optical density at 600 nm (OD_600_) of ~1, washed twice in PBS, sonicated twice for 10 s, and resuspended in DMEM. The macrophages were infected at a multiplicity of infection (MOI) of 1, and plates were incubated at 37°C in 5% CO_2_ for 3 h to allow for bacterial uptake. Medium was then removed, and the wells were washed twice with warm PBS and made replete with 500 µl/well of DMEM containing 10% FBS and PLAM. At specific time points, medium was removed, the wells were washed once with PBS, and the macrophages were lysed using 0.0625% aqueous sodium dodecyl sulfate. The lysates were serially diluted and plated to determine CFU.

### Phage infection.

*M. tuberculosis* strains were grown at 37°C to an OD_600_ of approximately 0.7 to 1.0 in fully supplemented Middlebrook 7H9. The cultures (1 ml) were washed three times with mycobacteriophage (MP) buffer (50 mM Tris, 150 mM NaCl, 10 mM MgCl_2_, 2 mM CaCl_2_) and resuspended in 0.1 ml MP buffer. For visualization using a Nikon Eclipse Ti microscope, mycobacterial phages (10^9^ PFU/ml; 5 or 25 µl) were added to a bacterial suspension (0.1 ml), and the suspension was incubated at 37°C overnight. The cells were spun down, washed with PBS, resuspended in MP buffer (0.01 ml), and spread on a microscope slide. For visualization by scanning electron microscopy, the bacterial suspension (0.01 ml) was mixed with phage lysate (10^9^ PFU/ml; 0.03 ml) for 5 min prior to fixation (see below).

### Scanning electron microscopy.

The bacterium-phage lysate mix was diluted 1/1 with a fixative solution (2.5% glutaraldehyde, 0.1 M sodium cacodylate, 0.2 M sucrose, 5 mM MgCl_2_, pH 7.4). The samples were plated onto poly-l-lysine-coated coverslips, dehydrated through a graded series of ethanol, critical point dried using liquid carbon dioxide in a Tousimis Samdri 795 critical point dryer (Rockville, MD), and sputter coated with chromium in a Quorum EMS 150T ES (Quorum Technologies Ltd., United Kingdom). The samples were examined in a Zeiss Supra field emission scanning electron microscope (Carl Zeiss Microscopy, LLC, North America) using an accelerating voltage of 2 kV.

### Detection of the *M. tuberculosis* persister population using Φ^2^DRM9.

*M. tuberculosis* strains were grown as described for phage infection (see above), washed twice with MP buffer, and diluted to an OD_600_ of ≈0.1 in 7H9 medium without tyloxapol in 96-well plates. Each well containing 0.1 ml of culture was treated with or without INH (1 mg/liter) and incubated at 37°C for 2 days. Samples and untreated controls were infected with 0.1 ml of the phage Φ^2^DRM9 at an MOI of 10 for 16 h at 37°C. For each sample and control, 10,000 events were acquired on an S3e cell sorter (Bio-Rad, CA) after gating the singlets on forward scatter (FSC) A and side scatter (SSC) A on a log_10_ scale. The data were analyzed using the FlowJo software package (version 10.0.7; Tree Star, Inc., Ashland, OR) by gating for GFP^+^ and GFP^+^ tdTomato^+^ cells in comparison to uninfected cells. The RFP population was back-gated to determine GFP expression distribution in these cells.

### MIC determination.

Serial 2-fold dilutions of each drug were prepared in the inside wells of sterile 96-well plates for a final volume of 0.1 ml. The outside wells of the plates were filled with 0.2 ml PBS. The strains were grown to mid-log phase (OD_600_ of ≈0.8 to 1) and diluted 1/1,000, and 0.1 ml of diluted culture was then added to each inside well. The plates were incubated at 37°C for 7 days. OD_590_ was read on a Victor 3V plate reader (PerkinElmer, Waltham, MA), and the MIC was determined as the lowest concentration of drug that prevented growth. MIC data were confirmed by adding an aqueous solution of resazurin (0.03 ml; 0.2 mg/ml) to each well and further incubating the plates for 2 to 3 days. The MIC was determined as the lowest concentration of drug that prevented the conversion of resazurin (blue) to resorufin (pink).

### Isolation of INH- and RIF-resistant strains.

Cultures of mc^2^7901 and mc^2^7902 grown to an OD_600_ of ≈2 were plated on 7H10-OADC-glycerol-PLAM plates containing INH or RIF at a concentration of 1 mg/liter. The plates were incubated at 37°C for up to 8 weeks. INH- and RIF-resistant mutants were patched onto 7H10-OADC-glycerol-PLAM plates containing INH (0 and 1 mg/liter) or RIF (0 and 1 mg/liter), respectively, to confirm drug resistance. Following incubation for 4 weeks, one INH-resistant and one RIF-resistant mutant from mc^2^7901 and mc^2^7902 were then cultured in 7H9-OADC-glycerol-tyloxapol-PLAM to an OD_600_ of ≈2. The INH-resistant and RIF-resistant mutants were plated on Middlebrook 7H10-OADC-glycerol-PLAM plates containing RIF (1 mg/liter) or INH (1 mg/liter), respectively, and incubated at 37°C for up to 8 weeks. INH-RIF-resistant mutants were patched onto 7H10-OADC-glycerol-PLAM plates containing INH (0 and 1 mg/liter) or RIF (0 and 1 mg/liter) to check for drug resistance. The plates were incubated at 37°C for 4 weeks.

### Mouse infection.

C57BL/6 and SCID female mice (6 to 8 weeks old) were obtained from Envigo (Somerset, NJ). The animal protocol “Evaluation of the safety and the efficacy of attenuated mycobacterial vaccine vectors” used in this study was approved by the Einstein Animal Institute, which is accredited by the “American Association for the Use of Laboratory Animals” and accepts as mandatory the NIH “Principles for the Use of Animals.” The mycobacterial strains were grown to mid-log phase (OD_600_ of ~0.6 to 0.8), centrifuged, and washed twice with PBS containing 0.05% tyloxapol. Cell pellets were resuspended in PBS-tyloxapol, sonicated twice, and diluted in PBS-tyloxapol. SCID mice were infected via tail vein injection with 5 × 10^5^ CFU of each strain. C57BL/6 mice were infected via the subcutaneous route at a dose of 5 × 10^5^ CFU (control mice received 0.1 ml PBS subcutaneously). C57BL/6 mice were boosted 3 weeks later with another dose of 5 × 10^5^ CFU of each strain. Six weeks postimmunization, C57BL/6 mice were infected via low-dose aerosol (28) with H37Rv (48 CFU). For organ bacterial burden determination, mice were euthanized, and lungs and spleens were collected and homogenized in PBS-tyloxapol. The lung and spleen homogenates were plated on Middlebrook 7H10-OADC-glycerol and Middlebrook 7H10-OADC-glycerol-PLAM. CFU were counted after 4 and 8 weeks of incubation at 37°C. Colonies that grew on Middlebrook 7H10-OADC-glycerol-PLAM plates were picked and patched onto Middlebrook 7H10-OADC-glycerol and Middlebrook 7H10-OADC-glycerol-PLAM plates, and the plates were incubated at 37°C for 4 weeks to check for nutrient requirements.
